# Ex vivo detection of SARS CoV 2 spike protein in human hair follicles and potential link to telogen effluvium

**DOI:** 10.1038/s41598-025-28909-3

**Published:** 2025-11-26

**Authors:** Stefanie Klingenstein, Natalia Ruetalo, Peter Helmut Neckel, Alexander Kleger, Michael Schindler, Stefan Liebau, Moritz Klingenstein

**Affiliations:** 1 Institute of Neuroanatomy and Developmental Biology (INDB), University of Tübingen, Österbergstraße 3, 72070 Tübingen, Germany; 2 Institute for Medical Virology and Epidemiology of Viral Diseases, University Hospital Tübingen, Tübingen, Germany; 3 Institute of Clinical Anatomy and Cell Analysis, University Tübingen, Tübingen, Germany; 4 Institute of Molecular Oncology and Stem Cell Biology, Ulm University Hospital, Ulm, Germany; 5 Division of Interdisciplinary Pancreatology, Department of Internal Medicine I, Ulm University Hospital, Ulm, Germany; 6 Core Facility Organoids, Ulm University, Ulm, Germany

**Keywords:** ACE2, Hair loss, Human hair follicle (HF), Human plucked hair, Keratin (KRT), Keratinocytes, SARS-CoV-2, Telogen effluvium (TE), TMPRSS2, Virus entry proteins, Cell biology, Diseases, Immunology, Medical research, Molecular biology

## Abstract

Telogen effluvium (TE), a form of hair loss, has emerged in a subset of COVID-19 patients, typically occurring several weeks after infection. TE is characterized by premature entry of hair follicles (HFs) into the telogen phase of the hair cycle. We analyzed the expression of the viral entry factors ACE2 and TMPRSS2 in human scalp specimens. In cryosections of skin biopsies and plucked HFs, both proteins localized to the basal layer of the outer root sheath (ORS), with TMPRSS2 also present in Henle’s layer of the inner root sheath (IRS). Ex vivo infection of plucked HFs with SARS-CoV-2 revealed nucleocapsid (NC) protein immunoreactivity in the ORS in two of three donors, corresponding to the K15⁺ epithelial stem/progenitor cell population. These NC⁺ cells co-expressed cleaved caspase3 (CAS3) and TUNEL, indicating apoptosis-related changes. Our findings suggest that SARS-CoV-2 nucleocapsid protein accumulates in ORS cells, potentially triggering local stress responses and apoptosis. This may provide a mechanistic link between SARS-CoV-2 infection and TE and highlights the HF as a potential, yet understudied, target organ in COVID-19.

**Supplementary Information:**

The online version contains supplementary material available at 10.1038/s41598-025-28909-3.

## Introduction

COVID-19, caused by severe acute respiratory syndrome coronavirus 2 (SARS-CoV-2), has presented a wide array of health challenges. While respiratory symptoms, ranging from sore throat and coughing to respiratory failure and pneumonia-induced systemic inflammatory responses, dominate the acute phase of COVID-19^1^, chronic symptoms and long-term consequences of the disease came into focus later on. Amongst them are disorders of the gastrointestinal tract^2^, cardiac^3^, hepatobiliary^4^, or nervous system^5^. However, the symptomatic spectrum does not only affect internal organs, as the skin and its appendices were repeatedly reported to exhibit COVID-19-related changes, like redness, skin rash, or hair-associated symptoms^6–8^. Similarly, other viral infections, including Varicella zoster^9^ or Dengue fever^10^, are well-known to inflict skin-related symptoms, particularly hair loss, which in turn was also observed in infections with the SARS-CoV-2^11–19^.

The human hair is a complex organ consisting of different cell types with a highly regenerative capacity^20^. The main cell type found in HFs is the keratinocyte. Keratinocytes are specialized cells of the human epidermis and skin appendices and are characterized by the expression of various water-repellent keratin (KRT) molecules^21^. These KRTs are important for the assembly of structural fibrous proteins and provide stability and shape to the cells. The most common form of hair loss in humans, predominately affecting males after puberty, is the pattern or androgenetic hairlessness (alopecia) caused by genetically determined hypersensitivity to the male hormone 5α-dihydrotestosterone^22^. In contrast, diffuse hair loss, which causes hair thinning and loss across the head, affects both sexes at any age. Telogen effluvium (TE) is the most common form of diffuse hair loss and is caused by hair cells entering the telogen phase (resting phase of the hair cycle) at an early stage^23–25^.

This synchronization of HFs leads to an immediate exit from the anagen phase with active hair growth and is most commonly triggered by severe infections with high fever or systemic inflammation, followed by strong emotional or physical stress, major surgery or trauma, postpartum hormonal changes, medication use, and endocrine dysregulations such as thyroid dysfunctions. Nutritional deficiencies and other systemic conditions have also been described as potential contributors^26,27^. The classical symptom of hair shedding typically occurs three to four months after the triggering event has resolved^28^.

TE was previously described in COVID-19 patients^29–32^, and several reasons for the high number of telogen HFs are discussed, including the release of inflammatory cytokines, nutritional depletion, direct damage to hair cells, or—given the special circumstances during the pandemic—emotional stress^16^. Intriguingly, post-COVID-19 TE appears to occur earlier than classic virus-associated TE, with onset frequently within 56–74 days (2–3 months) after infection, and in some cases as early as 2–4 weeks^16,17,33,34^. Given the broad spectrum of systemic and local triggers, it has been hypothesized that SARS-CoV-2 may induce transient, stress-related responses within the HF epithelium itself, potentially contributing to early-onset or self-limited forms of TE.

During the 2002/2003 SARS-CoV-1 outbreak, Angiotensin-converting enzyme 2 (ACE2) was identified as the functional entry receptor for the virus, a finding that later proved central to understanding SARS-CoV-2 cell entry^35^. The main mechanism of SARS-CoV-2 also involves the virus binding to the ACE2 on the surface of human cells, followed by cleavage by the transmembrane protease serine 2 (TMPRSS2), facilitating the virus’s entry into the cell^36,37^. Current research focuses intensely on these prominent viral entry proteins, as they play crucial roles in the infection and pathogenesis of the virus.

To establish a potential link between SARS-CoV-2 infection and TE in human HFs, we analysed different hair tissue specimens and derivatives for the expression of both viral entry proteins ACE2 and TMPRSS2. ACE2 and TMPRSS2 are two prominent host cell surface proteins that facilitate SARS-CoV-2 entry, although alternative, TMPRSS2-independent routes have also been described^36,38^.

To investigate SARS-CoV-2-mediated effects on hair follicle integrity and potential TE pathogenesis, we infected freshly plucked HFs ex vivo with SARS-CoV-2 for 96 h and analyzed them together with skin-embedded HFs and keratinocytes for viral nucleocapsid protein, ACE2 and TMPRSS2 expression on RNA and protein levels. A histopathological case series by Mazeto et al. (2022) provided electron microscopy evidence of SARS-CoV-2 particles within the cytoplasm of ORS cells in patients with COVID-19-associated effluvium, supporting the notion of direct follicular involvement^39^.

In line with this, we detected nucleocapsid protein and apoptotic markers in ORS keratinocytes after prolonged virus exposure, further supporting the hypothesis of a direct viral effect on HF epithelial cells. These findings suggest that HFs may be directly infected by the virus, providing a possible mechanism for COVID-19-associated TE.

## Results

### Histological staining and HF marker expression of human hair specimen

The human hair organ is composed of a hair shaft and a hair root, which is sunk into the skin. The HF constitutes a complex functional unit composed of numerous cell types with varying degrees of differentiation. A prominent structure on plucked hair that can be seen even with the naked eye is the root sheath, supporting the hair root and thereby anchoring the hair in the skin^40^.

**Fig. 1:**
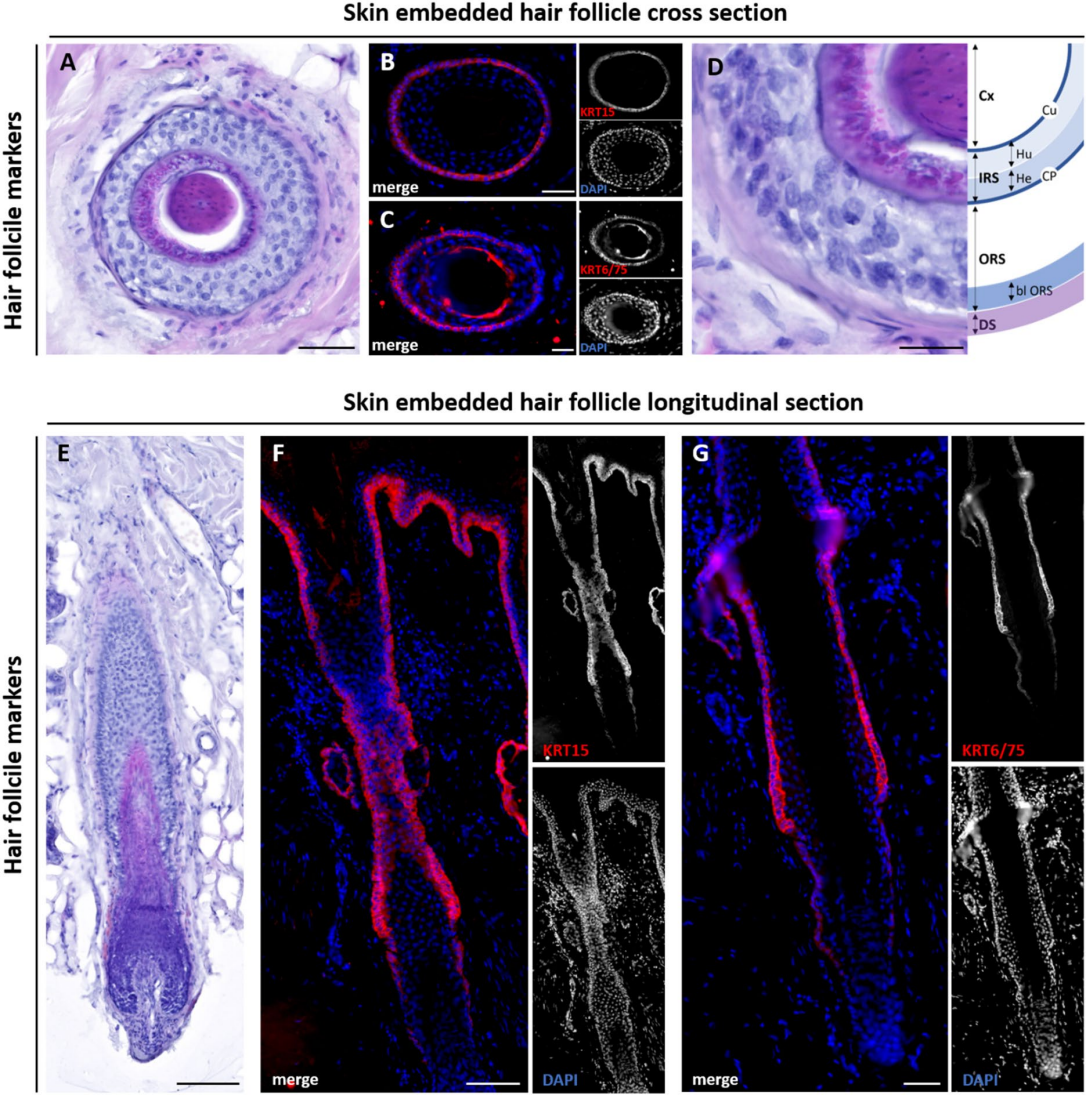
HE and IF staining in longitudinal and cross sections of skin-embedded HF. Cross section (**A**–**D**) and longitudinal section (**E**–**G**) of a skin-embedded hair follicle (HF). Histological haematoxylin-eosin (HE) staining of a skin-embedded HF (**A**,**E**) and marked schematic structures of the HF (**D**). The skin-embedded HF is surrounded by the dermal sheath (DS), a connective tissue layer, where capillaries and nerve terminals are located. In the outer root sheath (ORS), a single-layered basal layer (bl) can be distinguished. The inner root sheath (IRS) consists of the companion layer (CP), Henle’s layer (He), and Huxley’s layer (Hu). Towards the cortex (Cx), the IRS is delimited by the cuticular layer (Cu). Immunofluorescence staining of keratin (KRT) 15 (**B**,**F**, red) and KRT6/75 (**C**,**G**, red). KRT15 is a specific marker for the basal ORS. HF stained with antibody against KRT6/75 show two distinct layers, namely the suprabasal layer of the ORS and the companion layer. Nuclear marker DAPI is shown in blue. Scale bar: 100 μm (**A**,**E**), 50 μm (**B**,**C**,**D**,**F**,**G**).

For better visualization of the basic histology of the hair organ, we applied haematoxylin & eosin (HE) staining on transversal and longitudinal sections. A transverse HE section illustrates the circular arrangement of the different follicular layers (Fig. 1A). In the outer root sheath (ORS), also called hair root, a single-layered basal cell layer can be distinguished, which is in continuity with the basal layer of the multi-layered epithelial tissue into the hair funnel. The basal ORS is marked by KRT15 immunoreactivity (Fig. 1B), whereas suprabasal ORS cells and the companion layer can be visualized by KRT6/75 staining (Fig. 1C). The HF consists of an inner and outer epithelial root sheath. The ORS forms a cover around the inner root sheath (IRS), which is demarcated by a small layer, the companion layer. The IRS, in turn, encloses the hair shaft and descends from matrix cells located on the outer edge of the hair bulb. The IRS can be further subdivided into three defined layers: The outermost layer is Henle’s layer (He), consisting of one single layer of cubic cells with a flattened or even no visible nuclei. The Huxley’s layer (Hu) is the middle layer and comprises bigger cells with oval or flattened nuclei. The cuticula (Cu) builds the innermost layer of the IRS and lies in the immediate vicinity of the hair shaft (Fig. 1D)^41^.

In a longitudinal overview, the HE staining illustrates the HF embedded in dermal tissue (Fig. 1E). The HF is surrounded by a thin connective tissue layer, the dermal sheath, embedding numerous blood capillaries, sensitive nerve terminals, and adipocytes^42^. Numerous small blood capillaries within the dermal sheath supply the HF with nutrients, oxygen, and hormones^42^.

In order to explain the progression of hair loss after COVID-19 disease, the detection of individual layers of HF is important to pinpoint the location of TMPRSS2 and ACE2. For this purpose, longitudinal and cross sections of HF were stained with specific keratins that are expressed only in certain layers of the HF. KRT15 is a well-established marker of the basal cell layer (bl) of the ORS (Fig. 1F)^43^. In contrast, in a longitudinal section, KRT6 and KRT75 stain the suprabasal layer of the ORS and the companion layer, respectively, as previously described^44–46^ (Fig. 1G).

#### ACE2 protein expression in cross and longitudinal sections of HF

**Fig. 2:**
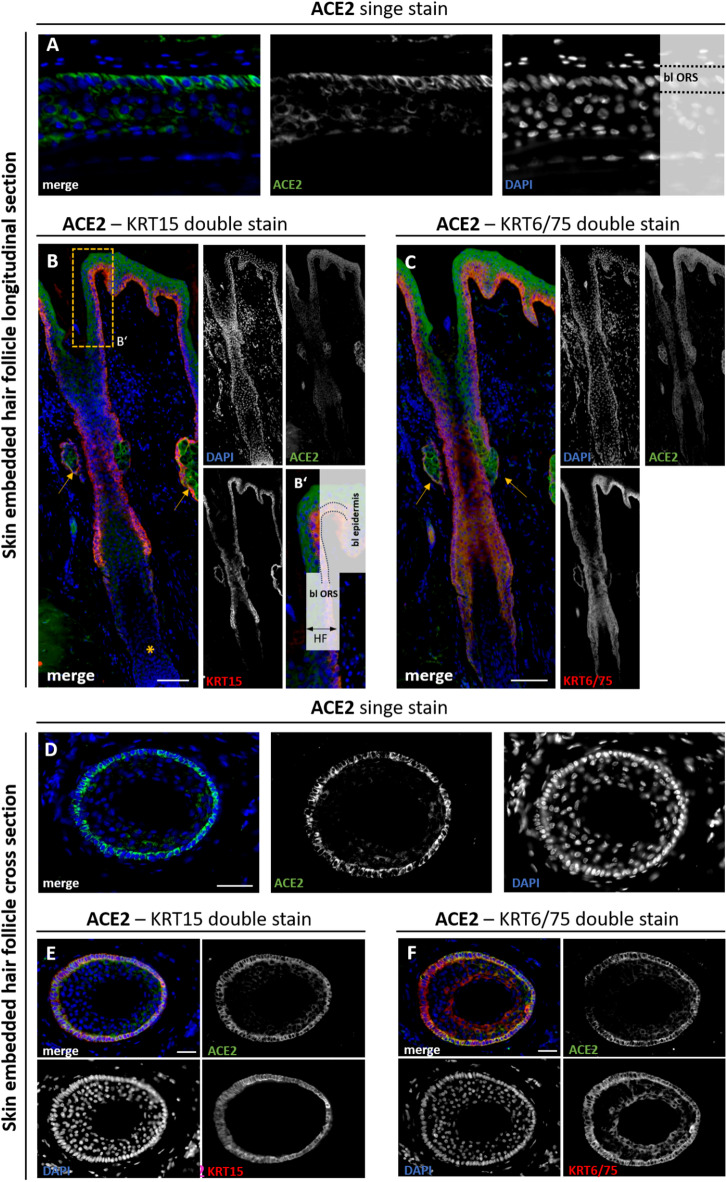
ACE2 staining in longitudinal and cross sections of skin-embedded human HF. Single ACE2 (green) expression in a longitudinal section of skin-embedded HF, showing strong signal in the basal layer of the outer root sheath (bl ORS) (**A**). Longitudinal section co-stained for ACE2 (green) and the ORS-specific marker KRT15 (red) (**B**). ACE2 expression is confined to the basal layer (bl) of the ORS and the basal layer of the epidermis. Yellow arrows mark sebaceous glands, the hair papilla is indicated by a yellow asterisk. Magnified view (**B’**) of the boxed area in (**B**), illustrating the ACE2-positive basal ORS and its continuity with the epidermal basal layer. Longitudinal section co-stained for ACE2 (green) and the ORS/companion-layer marker KRT6/75 (red) (**C**). ACE2 signals partially overlap with KRT6/75 in the basal ORS and the companion layer, yellow arrows indicate sebaceous glands. Single ACE2 staining in a cross section of skin-embedded HF, showing signal in the basal ORS and Henle’s layer (He) of the inner root sheath (IRS) (**D**). Cross section co-stained for ACE2 (green) and KRT15 (red), confirming ACE2 localization to the basal ORS (**E**). Cross section co-stained for ACE2 (green) and KRT6/75 (red), revealing ACE2 expression in both the basal ORS and the companion layer (CP), located between the ORS and IRS (**F**). Nuclear marker DAPI is shown in blue. Scale bar 100 μm (**B**,**C**), 50 μm (**A**,**D**–**F**).

To assess the distribution of ACE2 in human HFs, we analyzed both skin-embedded parietal scalp tissue and freshly plucked HFs by immunofluorescence. Both sample types revealed a comparable expression pattern of ACE2 in distinct HF compartments. For visualization, we selected representative images from both preparations, primarily based on signal clarity and tissue integrity. Single ACE2 staining of skin-embedded HFs showed a strong signal exclusively in the basal layer of the ORS (Fig. 2A). This specific location is characterized by a single-layered, prismatic cell layer harbouring a stem cell niche for various cells of the HF^47^. In the skin, ACE2 can be detected in all layers of the epidermis, except the uppermost layer, the stratum corneum^48^. In longitudinal sections, the fibrous dermal sheath and adjacent sebaceous glands were visible, with sebaceous glands showing strong ACE2 positivity (yellow arrows, Fig. 2B). Sweat glands were also strongly positive for ACE2 (Supplemental Figure S1 A, B), as previously observed for other gland cells, such as nasal glands or Bowman’s glands in the respiratory and olfactory epithelium^49^. Panel B′ shows a magnified view of the boxed area in B, illustrating the ACE2-positive basal ORS and its direct continuity with the basal layer of the epidermis. Co-staining with ORS-specific markers KRT14 and KRT15^50–52^ confirmed the localization of ACE2 to the outermost layer of the ORS (Fig. 2B, Supplemental Figure S1 C). KRT75 is a well-known marker for the companion layer of the IRS, and co-staining with KRT6/75 revealed signals not only in this layer but also in the basal layer of the ORS (Fig. 2C)^53^. The basal layer of sebaceous glands was likewise positive for KRT15 and KRT6/75 (arrows in Fig. 2C). In cross-sections, single ACE2 signals were consistently confined to the basal ORS, a localization that was verified by co-staining with the basal epithelial marker KRT15 (red) (Fig. 2D, E). In cross sections, co-staining with KRT6/75 confirmed ACE2 signals in the companion layer of the IRS and additionally revealed ACE2 expression in the basal layer of the ORS (Fig. 2F).Taken together, our co-staining of ACE2 with KRT14, KRT15, and KRT6/75 exhibited the highest immunoreactivity of ACE2 in the basal layer of the ORS.

#### TMPRSS2 protein expression in cross and longitudinal sections of HFs

**Fig. 3:**
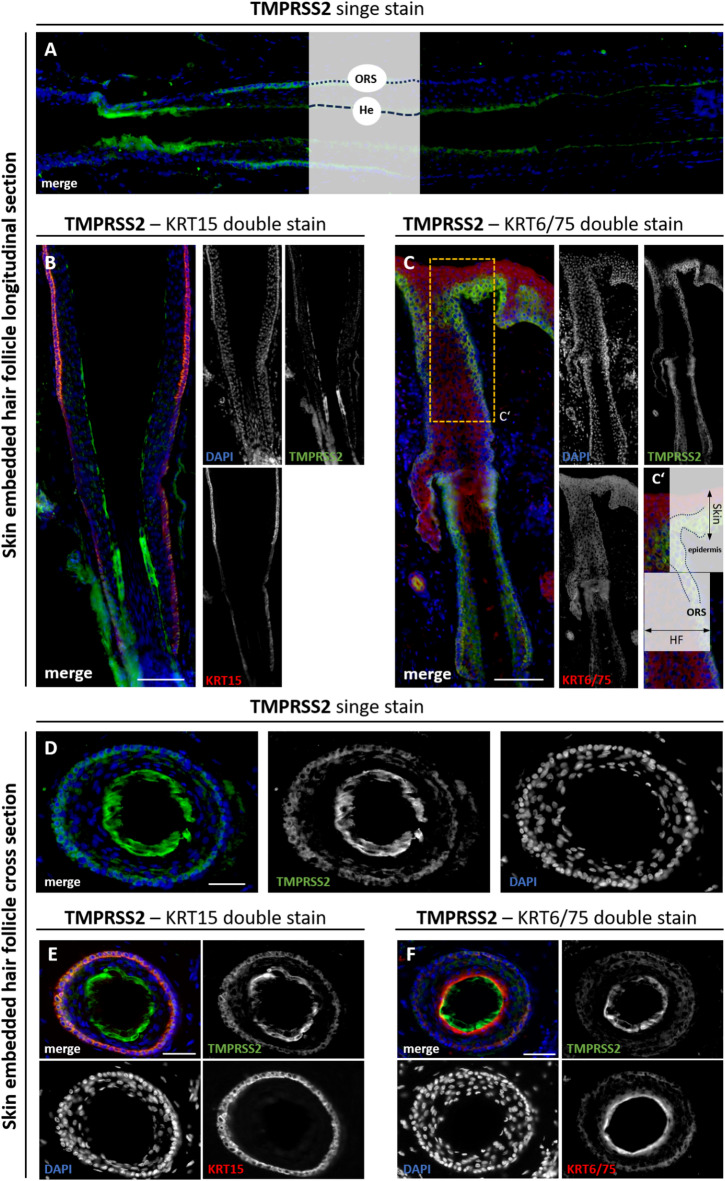
TMPRSS2 staining in cross and longitudinal sections of plucked and skin-embedded human HF. Single TMPRSS2 (green) staining in a longitudinal section (**A**) of skin-embedded HF showing strong signal in the outermost cell layer of the papillary region adjacent to the dermal sheath and weaker expression within the outer root sheath (ORS). Co-staining of TMPRSS2 (green) with the ORS marker KRT15 (red) in a longitudinal section (**B**) confirms TMPRSS2 localization in the basal and suprabasal layers of the ORS. Double labeling of TMPRSS2 (green) with the companion-layer marker KRT6/75 (red) allows clear distinction between ORS and inner root sheath (IRS) compartments (**C**). Magnified view (**C’**) of the boxed area in (**C**) highlighting partial overlap of TMPRSS2 and KRT6/75 signals at the interface between the ORS and the companion layer (CP) as well as the orientation relative to the epidermis. Cross section (**D**) showing TMPRSS2 expression in the ORS and Henle’s layer (He) of the IRS. Cross section co-stained for TMPRSS2 (green) and KRT15 (red) confirming TMPRSS2 localization in the basal ORS (**E**). Cross section (**F**) co-stained for TMPRSS2 (green) and KRT6/75 (red) delineating the boundary between TMPRSS2-positive ORS cells and the companion layer with partial overlap at the interface. (Nuclear marker DAPI is shown in blue. Scale bars: 100 μm (**B**,**C**), 50 μm (**A**,**D**–**F**).

Many cell layers change their alignment and orientation in the transition area between the papillary region and the ORS. For the precise determination of TMPRSS2 protein expression in HF specimens, we examined both skin-embedded HFs (Fig. 3, Supplemental Figure S1 B, D), which contain all skin appendages, such as sebaceous glands, sweat glands, smooth muscle cells, and adipocytes, and freshly plucked HFs (Fig. 3). In longitudinal sections of skin-embedded HFs, TMPRSS2 expression showed a strong signal in the outermost cell layer of the papillary region, adjacent to the dermal sheath (Fig. 3A, Supplemental Figure S1 E). Within the ORS, a weaker but still distinguishable TMPRSS2 signal was detected. The specific expression of TMPRSS2 in the ORS was verified by co-staining with the ORS-specific markers KRT15 and KRT14 (Fig. 3B, Supplemental Figure S1 B). Double labeling with KRT6/75 (red), a marker for the companion layer (CP), allowed clear distinction between inner and outer root sheath compartments (Fig. 3C). TMPRSS2 signals were confined to the ORS and partially overlapped with KRT6/75 at the ORS/CP interface, as illustrated in the magnified region (Fig. 3C’). In cross section, TMPRSS2 expression was most prominent in the outer cell layers of the follicle, corresponding to the ORS and the Henle’s layer (He) of the inner root sheath (IRS) (Fig. 3D). Co-staining with KRT15 (red) in cross sections confirmed TMPRSS2 localization in the basal ORS (Fig. 3E). Co-staining with KRT6/75 (red) delineated the boundary between TMPRSS2-positive ORS cells and the companion layer, with partial overlap at the interface (Fig. 3F). Additionally, histological staining (Supplemental Figure S1 F, F’) and fluorescence with the endothelial marker *Ulex Europaeus Agglutinin* (UEA1) confirmed the presence of capillaries in the dermal sheath directly adjacent to the ORS (Supplemental Figure S1 G–G’’).

**Fig. 4:**
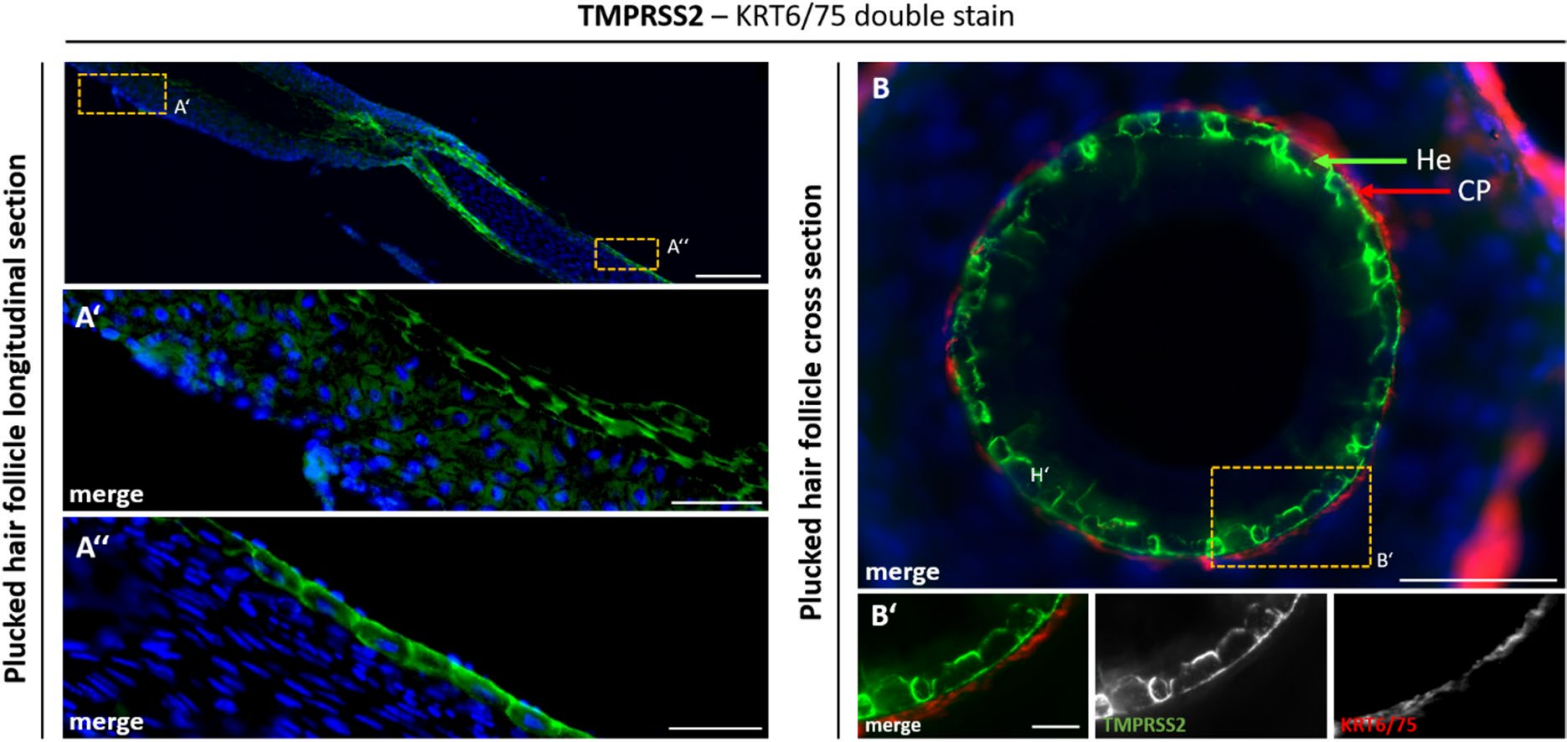
Layer-specific TMPRSS2 localization in human hair follicles revealed by co-staining with KRT6/75. TMPRSS2 (green) expression in longitudinal (**A**–**A**″) and cross (**B**–**B**′) sections of human hair follicles (HFs). Single TMPRSS2 (green) expression in a longitudinal (**A**–**A’’**) section of plucked HFs. The highlighted magnified section in (**A’**) shows the expression of TMPRSS2 in the area of the outer layer of the ORS. The highlighted magnified section in (**A’’**) shows the expression of TMPRSS2 in the area of the hair bulb. There is a clear expression in the outermost layer of the hair bulb. Co-staining of TMPRSS2 (green) and ORS/companion layer-specific marker KRT6/75 (red) in a cross (**B**) section of plucked HFs. The highlighted magnified section in (**B’**) shows the expression of TMPRSS2 in the He, which is a part of the IRS and directly connects to the companion layer (CP) inwards. Nuclear marker DAPI is shown in blue. Scale bar 200 μm (**A**), 50 μm (**A’**, **A’’**, **B**), 10 μm (**B’**).

In plucked HFs, TMPRSS2 signals were again observed in the ORS (Fig. 4A, A’, A’’). At the boundary between ORS and IRS, a strong and highly specific TMPRSS2 signal was localized to the IRS (Fig. 4B, B’). Guided by KRT6/75 demarcation of the companion layer, this signal could be assigned specifically to Henle’s layer (He) of the IRS. The IRS links the ORS to the hair shaft and consists of the companion layer, Henle’s layer, and Huxley’s layer. Differential interference contrast (DIC) images revealed characteristic companion layer cells (referred to as “Flügelzellen” in German histological literature) within the Huxley layer that extend into Henle’s layer, aiding in the identification of inner root sheath sublayers^54^. Henle’s layer itself is composed of keratinized cubic cells with flattened nuclei.

By combining keratin markers and morphological analysis, we were able to distinguish individual HF layers and pinpoint TMPRSS2 expression in specific compartments, such as Henle’s layer and the outermost papillary region.

### Immunofluorescence staining of ACE2 and TMPRSS2 in primary keratinocytes

As we could detect a specific and definable expression pattern of ACE2 and TMPRSS2 in cross and longitudinal sections of HF specimens as well as in various layers of the multilayered, keratinized squamous epithelium of the skin, we next analysed the expression of the host entry factors in isolated, primary keratinocytes, which are the predominant cell type in the hair organ. Primary keratinocytes can be easily generated from plucked hair specimen under adherent culture conditions^55^. In line with the histological organization of the human hair follicle, these keratinocytes originate from the outer root sheath (ORS), which consists predominantly of keratinocytes. While not specifically labeled in the previous figures, the ORS layers shown in our histological stainings largely represent keratinocyte populations. First-appearing keratinocytes grow out from the distal part of the ORS and spread over the whole culture vessel within some days.

**Fig. 5:**
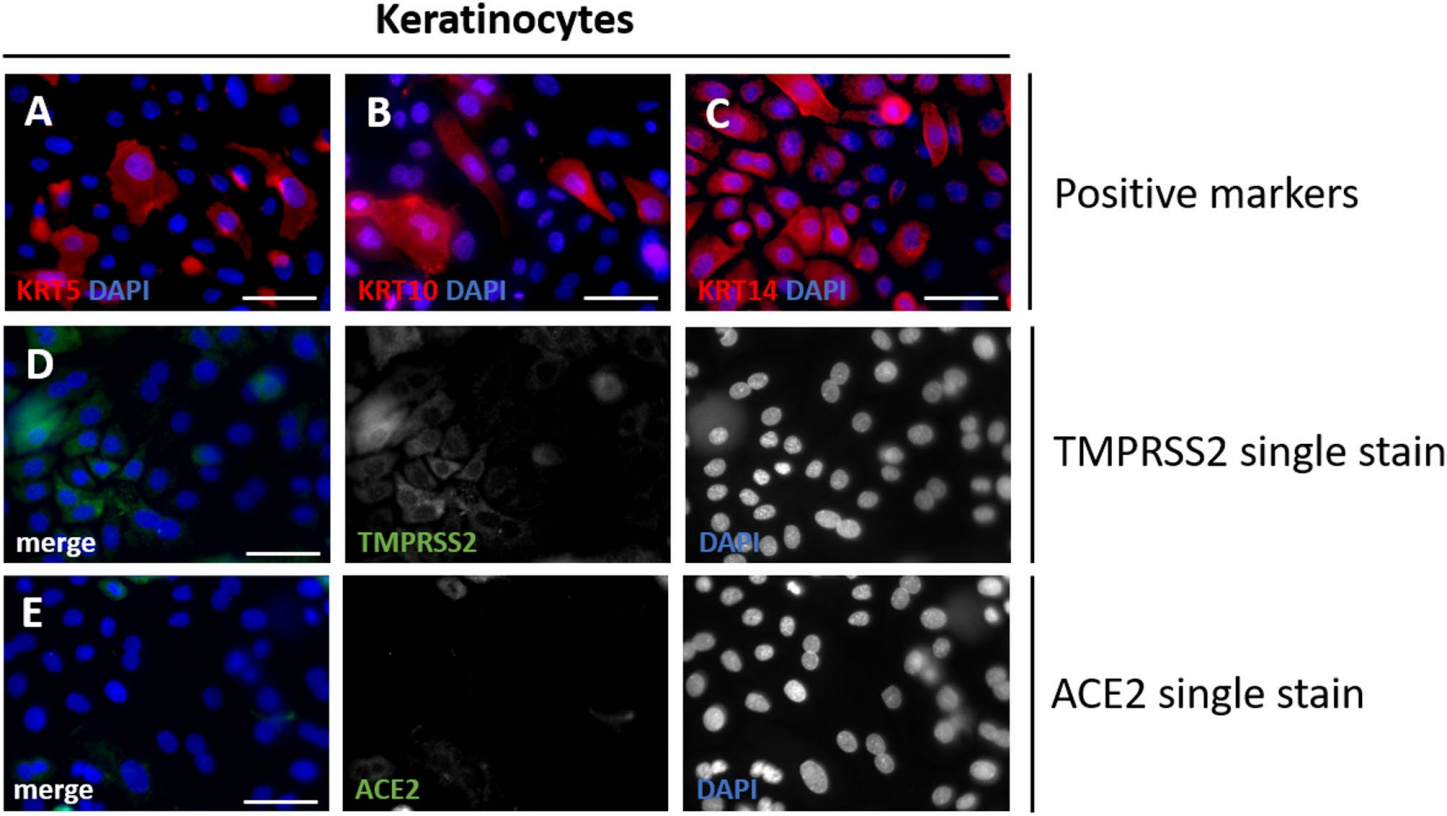
Staining of primary keratinocytes. Positive staining for adherent keratinocytes isolated from plucked human HFs KRT5 (**A**, red), KRT10 (**B**, red), and KRT14 (**C**, red). Primary keratinocytes were growing from plucked human hair follicles (HFs) under adherend culture conditions. Cells were stained against viral entry proteins TMPRSS2 (**D**, green) and ACE2 (**E**, green). Nuclear marker DAPI is shown in blue. Scale bar: 50 μm (**A**–**E**).

The keratin family are structural fibrous proteins that provide cell stability and shape^56^. As they are needed for the formation of intermediate filaments, keratins are located in the cytoplasm of keratinocytes^57^. Analysis of these isolated cells shows the expression pattern of several keratinocyte-specific keratins, like KRT5, KRT10, and KRT14 (Fig. 5A–C). In addition to keratin expression, primary keratinocytes derived from plucked HFs also showed weak TMPRSS2 signals in a subset of cells (Fig. 5D). For ACE2, no specific signal was detected (Fig. 5E). This observation aligns with previous reports describing low or absent ACE2 protein levels in cultured keratinocytes, which may reflect culture-related downregulation or a loss of differentiation-dependent expression.

### RNA expression of ACE2 and TMPRSS2 in plucked HFs and keratinocytes

**Fig. 6:**
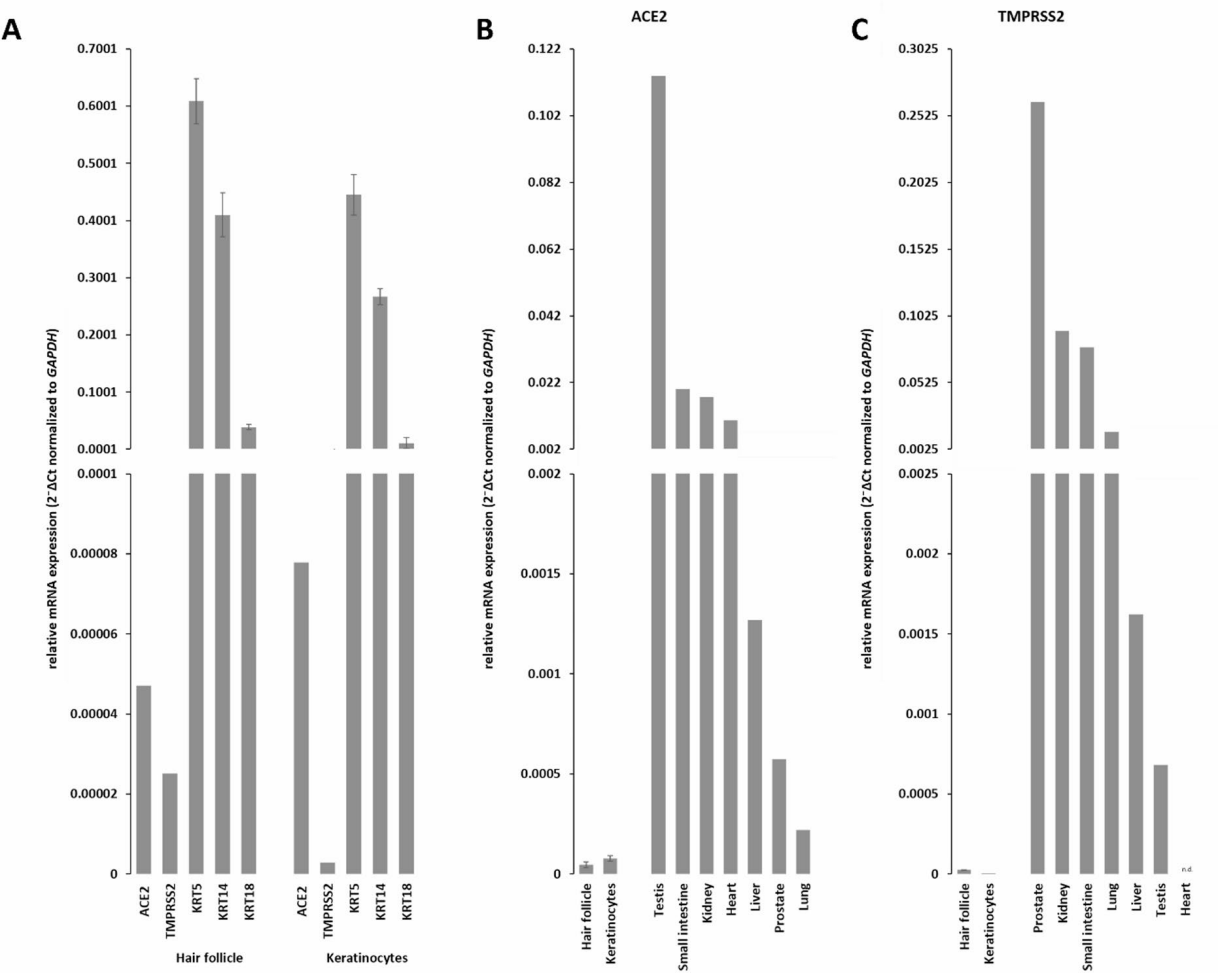
RNA expression profile. (**A**) Quantitative RNA expression profile of *ACE2*, *TMPRSS2* and three keratin probes (*KRT5*, *KRT14*, *KRT18*) in plucked hair follicles and primary keratinocytes. (**B**) Expression values represent mean 2⁻ΔCt ± SD normalized to GAPDH (*n* = 3 biological replicates, each measured in technical duplicates). Due to the linear scale of the y-axis, the comparatively low ACE2 and TMPRSS2 expression levels appear close to the baseline. *ACE2* mRNA level in both analysed specimens, as well as in selected human organs with high *ACE2* expression (small intestine, testis), moderate *ACE2* expression (kidney, heart, liver) and low *ACE2* expression (liver, prostate, lung). (**C**) *TMPRSS2* expression in both analysed specimens, as well as in selected human organs with high *TMPRSS2* expression (prostate, small intestine), moderate *TMPRSS2* expression (kidney, lung, liver) and low *TMPRSS2* expression (testis, heart). Number of single, independent experiments *n* = 3 (hair follicle, keratinocytes), *n* = 1 (testis, small intestine, kidney, heart, liver, lung, prostate), n.d. (not detected).

We next analysed the RNA level of *ACE2* and *TMPRSS2* in plucked HF samples and isolated keratinocytes. Three keratin probes (*KRT5*, *KRT14*, *KRT18*) were used as positive control for the plucked HFs and the cultivated keratinocytes (Fig. 6A). KRT5 and KRT14 are basal epithelial keratins expressed in the outer root sheath and in the basal layer of the epidermis, whereas KRT18 marks simple and glandular epithelia and can be detected in specific inner compartments of the hair follicle^58^. As expected, a high expression of all three keratins could be detected within the HFs and keratinocyte samples. ACE2 and TMPRSS2 mRNA were also detectable in both samples, although at markedly lower levels compared to keratins. Expression values represent mean 2⁻ΔCt ± SD normalized to GAPDH (*n* = 3 biological replicates). Quantitatively, ACE2 and TMPRSS2 were approximately 10²–10⁴-fold and 10³–10⁵-fold lower, respectively, than the keratin control genes KRT5, KRT14, and KRT18. Notably, ACE2 expression levels were consistently higher than TMPRSS2 in both tissue and cell culture (Fig. 6A). Notably, ACE2 expression levels were higher than TMPRSS2 in both tissue and cell culture (Fig. 6A).

For comparison, the relative RNA expression of ACE2 and TMPRSS2 was also measured in organs known to express different amounts of both genes (Fig. 6B, C). Based on published data, high ACE2 expression is expected in the human small intestine and testis, moderate expression in kidney, gall bladder, heart and liver, and low expression in liver, prostate and lung^59–61^. Our ACE2 data largely fit these reported patterns, except for the human testis, where we observed the highest ACE2 signal among the analysed organs. Even compared to organs with low ACE2 expression such as prostate or lung, the ACE2 levels in plucked HFs (4.7 × 10^−5^) and keratinocytes (7.8 × 10^−5^) were substantially lower. For TMPRSS2, high RNA levels are published for prostate and small intestine, which correlate with our data, showing strongest TMPRSS2 expression in prostate (2.6 × 10^−1^), followed by kidney (9.1 × 10^−2^) and small intestine (7.8 × 10^−2^). In contrast, liver (1.6 × 10^−3^) and lung (1.5 × 10^−2^) showed only moderate, and testis and heart very low expression levels^62–64^. In plucked HFs (2.5 × 10^−5^) and keratinocytes (2.8 × 10^−6^), TMPRSS2 expression was several orders of magnitude lower than in all other tested human organs (Fig. 6C). Because organ data were derived from single biological samples (*n* = 1), they are presented descriptively without statistical testing.

In the heart specimen, no *TMPRSS2* mRNA could be detected (n.d.). Furthermore, we were interested whether the sex of the HF donor had an influence on the RNA expression level and therefore analysed male (♂) and female (♀) plucked HFs (Supplemental figure S1 K). It was possible to detect *ACE2* and *TMPRSS2* expression in HFs from both donors, but no significant difference in the expression levels of the two sexes could be detected. The only exception was *KRT5*, which was significantly higher expressed in female HFs (*n* = 3, unpaired two-tailed TTEST *p* < 0,05).

### SARS-CoV-2 infection of plucked HFs

**Fig. 7:**
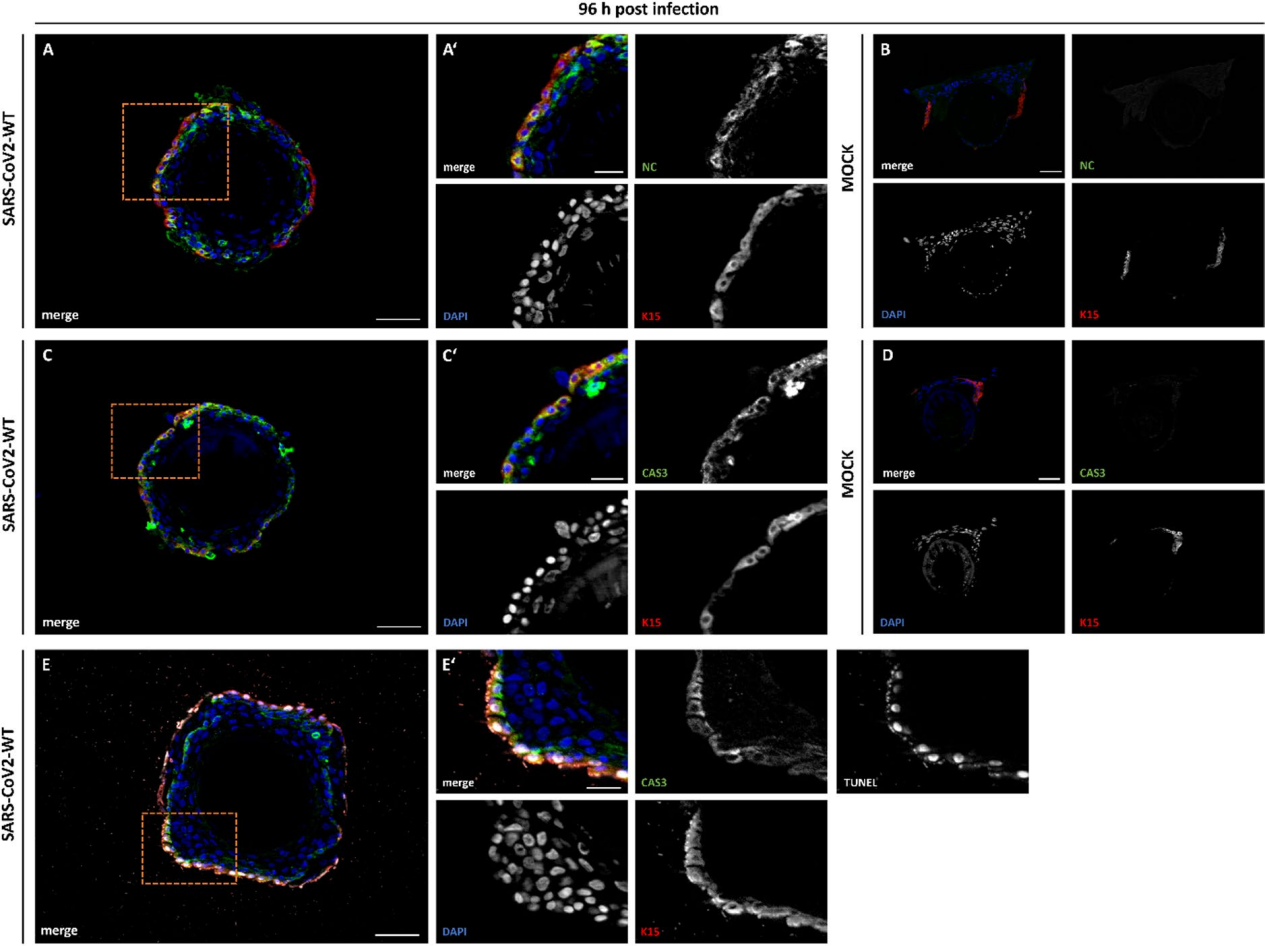
SARS-CoV-2 infection of plucked HFs. Nucleocapsid (NC, green) and keratin 15 (K, red) expression in SARS-CoV-2-WT (**A**) and Mock-infected (**B**) hair follicles. Main NC and K15 expression can be detected in the ORS only in virus infected HF, visible in the highlighted magnification (**A’**). Caspase3 (CAS3, green) and keratin 15 (K, red) expression in SARS-CoV-2-WT (**C**) and MOCK (**D**) infected hair follicles. Main CAS3 and K15 expression can be detected in the ORS only in virus infected HF, visible in the highlighted magnification (**C’**). Nucleocapsid (NC, green), keratin 15 (K, red) and TUNEL (white) expression in SARS-CoV-2-WT (**E**) infected hair follicles. Main CAS3, K15 and TUNEL expression can be detected in the ORS, visible in the highlighted magnification (**E’**). Nuclear marker DAPI is shown in blue. Scale bar: 50 μm (**A**–**H**), 20 μm (**A’**–**G’**).

To investigate whether plucked human HFs are permissive to virus infection, we incubated them ex vivo with a clinical SARS-CoV-2 isolate (lineage B.1) for 48 and 96 hours. Morphological analysis showed that the cells remained viable and continued to proliferate after infection, as primary keratinocytes grew out from the HFs (data not shown). This was observed both in cell exposed and non-exposed to the virus. In two out of three biological replicates, immunofluorescence analysis revealed positive signal for the viral nucleocapsid (NC) protein in the outermost, K15-positive layer of the follicle (Fig. 7A, A’), at 96 h post infection (hpi). No NC signal was observed in mock-treated controls (Fig. 7B). The NC + compartment corresponds to the basal layer of the ORS, previously shown to express both ACE2 and TMPRSS2. Also, in the exposed HFs, ACE2 was strongly detectable in the ORS (Supplemental Figure S2 A), while TMPRSS2 was present in the ORS and showed its strongest expression in Henle’s layer of the IRS (Supplemental Figure S2 B). To determine whether viral exposure might induce stress-related cellular changes, we stained for cleaved CAS3 and performed a TUNEL assay. In NC-positive follicles, cells in the outermost ORS layer co-expressed K15 and showed signal for cleaved CAS3 (Fig. 7C, C’) compared to mock controls (Fig. 7D). Moreover, TUNEL staining confirmed apoptotic changes in K15-positive ORS cells of infected HFs (Fig. 7E, E’), which were absent in mock controls (Supplemental Figure S2 D).

Taken together, our findings suggest that SARS-CoV-2-exposed HFs can exhibit NC localization in K15-positive cells of the ORS, accompanied by apoptotic changes. These results point to a possible stress-mediated reaction of outer HF cells upon extended exposure to viral components and may hint to infection of HFs with SARS-CoV-2, although our data do not prove productive infection.

## Discussion

### Localization of SARS-CoV-2 entry proteins ACE2 and TMPRSS2 in human hair follicles and keratinocytes

**Fig. 8:**
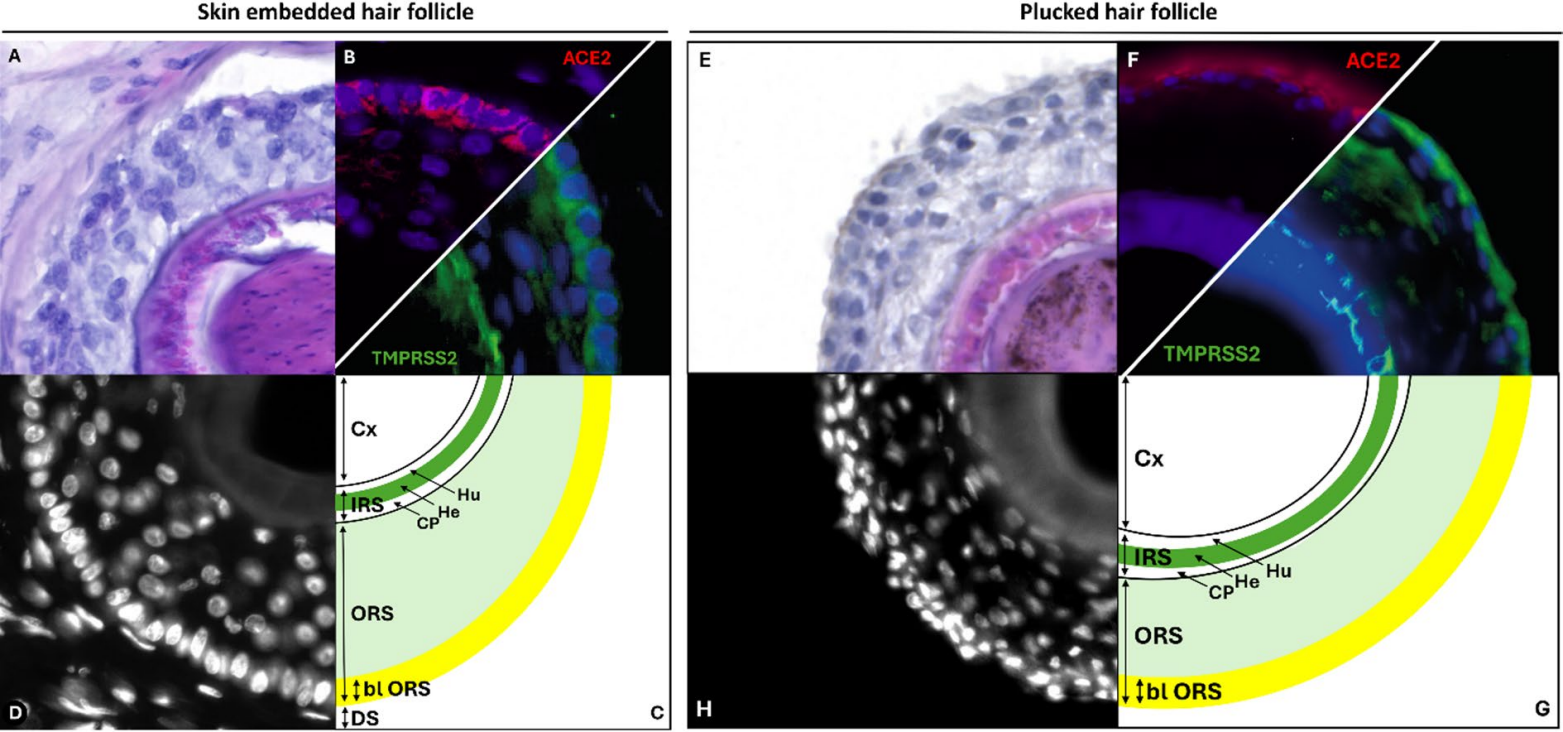
Schematic summary of ACE2 and TMPRSS2 staining of skin embedded and plucked human HFs. Schematic summary of the human skin embedded and plucked HFs. Histological HE staining (**A**,**E**), single nuclear DAPI staining (white, **D**,**H**) and ACE2, TMPRSS2 (green, **B**,**F**) staining. In the scheme (**C**,**G**) the ORS shows outward a single basal layer (bl ORS). Overlapping expression of ACE2 (red) and TMPRSS2 (green) within the basal layer (bl) of the ORS is indicated in yellow, corresponding to the color overlap observed under the fluorescence microscope. Next to the cortex (Cx), the inner root sheath (IRS) consists of the companion layer (CP), the Henle’s layer (He) and the Huxley layer (Hu). Note the dermal sheath (DS), where capillaries and nerve terminal are located, as well as the dermis, is missing in the plucked HF.

The aim of this study was to characterize the expression of the two main SARS-CoV-2 entry factors, ACE2 and TMPRSS2, in human hair follicles (HFs) and keratinocytes, thereby providing a molecular basis for the proposed link between COVID-19 and hair loss. Using both skin-embedded and freshly plucked scalp HFs, we were able to localize ACE2 and TMPRSS2 on RNA and protein level in specific cell layers of the ORS. While the overall expression pattern was similar across both specimen types, plucked HFs showed partial disruption of surrounding tissue layers due to mechanical forces during sampling (Fig. 8A–G). Figure 8 schematically summarizes the localization patterns, with ACE2 (red) and TMPRSS2 (green) expression and their partial overlap in the basal ORS indicated in yellow. Structures like sebaceous glands, sweat glands and the arrector pili muscle, which are still attached in skin punches, are typically absent in plucked HFs.

Co-staining with well-characterized ORS markers (KRT14, KRT15, KRT6/75) allowed us to assign ACE2 expression specifically to the basal layer of the ORS, which represents a prolongation of the *Stratum basale* of the epidermis. TMPRSS2, in contrast, was found throughout the ORS and also strongly expressed in the Henle’s layer of the inner root sheath (IRS), clearly distinguishable from the KRT6/75-positive companion layer. This layer-specific expression was consistently observed in both skin-embedded and plucked HFs and supports the notion that outer epithelial HF compartments provide the necessary molecular prerequisites for SARS-CoV-2 entry. These findings are in line with earlier studies that localized ACE2 expression to the basal epidermal layers in human skin, including the HF epithelium^65^.

In addition, we confirmed gene expression of both entry factors in plucked HFs and in primary keratinocytes derived from them. On the transcript level, ACE2 and TMPRSS2 were both detectable, though at lower levels in cultured keratinocytes compared to intact HFs and internal organs. On the protein level, ACE2 could not be detected in cultured keratinocytes, while TMPRSS2 showed weak signals in a subset of cells. These differences may reflect the impact of in vitro culture conditions, as primary keratinocytes are known to lose proliferative capacity and undergo terminal differentiation during outgrowth. This could contribute to the reduced or absent protein expression observed in cultured cells. Together, these data support that the outermost epithelial cells of the ORS – particularly those expressing KRT15 – form a potential entry zone for SARS-CoV-2 in the human HF.

### Detection of SARS-CoV-2 nucleocapsid protein in the outer root sheath of human hair follicles

Our findings provide initial ex vivo evidence for the presence of SARS-CoV-2 nucleocapsid protein (NC) in the ORS of human hair follicles (HFs) exposed to SARS-CoV-2, specifically in the KRT15 positive basal layer. This cell layer also expresses the viral entry factors ACE2 and TMPRSS2, suggesting a local permissivity for viral uptake. Importantly, immunoreactivity for NC was observed in two out of three biological replicates following 96 h of incubation with SARS-CoV-2 particles. In the third donor, however, NC was absent, and no viral RNA could be detected in the ORS suggesting variability in viral uptake or protein accumulation.

In parallel, primary keratinocytes grew out from both mock- and virus-incubated follicles and maintained their proliferative capacity, suggesting that SARS-CoV-2 exposure did not induce overt cytotoxicity. While we did not observe a consistent RT-qPCR signal in the current setting, these data are not sufficient to conclusively determine the presence or absence of productive viral replication. Nevertheless, the detection of NC at 96 hpi in two out of three donors—despite the lack of signal at 48 hpi—may reflect delayed viral protein accumulation, transient uptake, or abortive infection, as described for other non-cytopathic SARS-CoV-2 target tissues such as endothelial or immune cells, which can show intracellular viral protein expression in the absence of infectious virus release^65–67^. Taken together, these findings indicate that SARS-CoV-2 nucleocapsid protein can be detected in the outermost cell layer of the ORS, which also expresses ACE2 and TMPRSS2. Despite viral exposure, the infected HFs maintained their structural integrity and proliferative capacity for at least 96 h, suggesting that viral protein presence alone does not necessarily impair HF vitality in the short term. This further supports the hypothesis that SARS-CoV-2 may interact with epithelial progenitor cells without inducing immediate cytopathic effects yet potentially initiating downstream stress responses relevant for TE pathogenesis.

### Apoptosis-related signals in SARS-CoV-2-exposed hair follicle cells

To investigate whether the presence of viral proteins in hair follicle cells is associated with apoptotic processes, we examined the expression of two established apoptosis markers—cleaved CAS3 and TUNEL positivity—96 h after viral exposure. Notably, both markers were detected in the same KRT15-positive outermost cell layer of infected HFs, where the SARS-CoV-2 nucleocapsid protein had also been localized. In contrast, mock-infected control samples showed no positivity for either CAS3 or TUNEL. No morphological alterations or apoptotic signals were noted in the dermal papillae. Apoptosis markers were confined to the outer root sheath keratinocytes, which correspond to the ACE2⁺/TMPRSS2⁺ compartment.

These findings suggest that SARS-CoV-2 exposure may trigger apoptosis-related responses in a subset of ORS cells expressing ACE2 and TMPRSS2. The simultaneous detection of caspase-3 and TUNEL positivity in defined cells supports the presence of apoptotic processes, potentially reflecting late-stage programmed cell death. Importantly, these changes appear to be localized and do not affect the overall structural integrity or proliferative capacity of the follicle within the observed 96-hour timeframe. Thus, our data support a model in which SARS-CoV-2 may induce selective cellular responses without causing immediate cytopathic effects at the tissue level. The presence of both CAS3 and TUNEL signals in KRT15 positive ORS cells corroborates previous observations that SARS-CoV-2 can trigger caspase-mediated apoptosis in non-cytopathic target cells. For instance, Koupenova et al. demonstrated CAS3 activation in SARS-CoV-2–exposed platelets without productive replication^66^, while Zhang et al. showed that viral proteins such as ORF3a can directly induce CAS3-dependent cell death in epithelial cells^67^. A comprehensive review also highlights that CAS3 is a common mediator of virus-induced apoptosis across multiple cell types^68^. The mechanism by which this apoptosis is triggered remains speculative. It may reflect a direct cellular stress response to viral uptake or protein accumulation or could result from indirect mechanisms such as cytokine exposure, local inflammation, or impaired nutrient exchange within the follicle microenvironment. The specificity of the apoptotic signal to the outermost ORS cells, which also express ACE2 and TMPRSS2, supports a localized, stress-associated cell death pathway.

These results suggest that SARS-CoV-2-exposed outer root sheath cells undergo localized, non-cytolytic apoptotic changes, potentially contributing to early catagen transition and hair loss symptoms.

### Proposed mechanisms linking SARS-CoV-2 exposure to follicular stress and hair loss

Based on our findings, we propose that SARS-CoV-2 may interact with the hair follicle (HF) via localized exposure of the outer root sheath (ORS), a compartment that expresses both ACE2 and TMPRSS2. These entry factors were consistently detected in the basal epithelial layer of the ORS—an area closely associated with the perifollicular microvasculature in skin-embedded HFs. Using the endothelial marker UEA1, we confirmed the presence of capillaries in the dermal sheath, directly adjacent to the ACE2⁺ ORS. While viremia is not uniformly observed in all COVID-19 patients and the isolation of infectious virus from blood remains rare^69,70^, viral RNA and proteins have been detected in the circulation. Therefore, hematogenous dissemination—albeit sporadic—could serve as a potential route for SARS-CoV-2 to access perifollicular structures.

Following viral exposure, we observed SARS-CoV-2 NC in K15⁺ ORS cells, along with cleaved CAS3 and TUNEL positivity—suggestive of apoptosis-related responses in epithelial progenitor cells. These findings raise the hypothesis that local interaction with SARS-CoV-2 antigens—with or without productive viral replication—may trigger cell stress and programmed cell death. Previous reports have linked SARS-CoV-2 infection to CAS3 activation in other epithelial tissues (e.g. lung, olfactory epithelium)^66,71,72^, supporting the concept of virus-associated stress responses even in the absence of cytopathic effects.

Taken together, these molecular and histological observations provide a plausible mechanism by which SARS-CoV-2 may directly impair HF integrity. Disruption of the ORS—either by apoptosis, altered signaling, or metabolic stress—may precipitate the collapse of the anagen phase and contribute to the onset of telogen effluvium (TE). Our data add experimental support to previously discussed mechanisms of COVID-19-associated hair loss, including systemic cytokine surges, fever-related fibroblast stress, psychological strain, perifollicular microvascular dysfunction, and oxidative damage.

However, it remains unclear whether the observed apoptosis is driven primarily by viral infection, immune signals, or secondary stress responses. Further research—particularly using in vivo models or HF organoids—will be needed to delineate the exact contributions of direct versus indirect mechanisms in the pathophysiology of post-COVID hair loss.

### Interindividual susceptibility and limitations of current findings

While our study provides first ex vivo evidence for the presence of SARS-CoV-2 proteins and apoptosis markers in human hair follicles, the data are descriptive and exploratory in nature. Quantitative analyses, such as colocalization metrics or statistical evaluation of TUNEL-positive cells, were not feasible due to the limited number and variable preservation of post-mortem human samples. Instead, we focused on reproducible qualitative patterns across biological replicates, which consistently pointed to stress-associated cellular responses in outer root sheath cells. This limitation has been acknowledged in the interpretation of our results and highlights the need for future studies using standardized sample sets and quantitative image analyses.

Although we detected ACE2, TMPRSS2, CAS-3, and SARS-CoV-2 nucleocapsid protein in the outermost layer of plucked human HFs—suggesting a possible entry route for the virus into follicular epithelial cells—only a subset of COVID-19 patients develop symptoms of telogen effluvium (TE). This variability is likely multifactorial and may involve both viral and host-specific parameters.

Our analysis revealed that ACE2 expression is strictly confined to the basal layer of the outer root sheath (ORS), whereas TMPRSS2 shows broader, albeit weaker, expression throughout the ORS and highest levels in the Henle’s layer of the IRS. While this study is the first to report TMPRSS2 expression in this compartment, the functional consequences remain speculative. It is conceivable that TMPRSS2 contributes to IRS maturation by processing precursor proteins or modulating growth factor activity. However, this does not directly explain the interindividual differences in TE manifestation and should rather be considered in the broader context of follicular homeostasis.

More likely explanations for the clinical heterogeneity include:

**Variability in viral load and tissue exposure**: Not all infections result in sufficient viral presence within the skin or follicular structures. Severe disease courses—especially those involving systemic inflammation or high viral burden—have been associated with a higher likelihood of TE, whereas mild or asymptomatic infections may not induce a strong enough stress response to affect hair cycle dynamics.

**Differences in immune response**: Individuals exhibit varying immunological reactions to SARS-CoV-2, which may influence local inflammatory signaling, tissue damage, and the induction of apoptosis within HFs.

**Systemic cofactors**: Psychological stress, fever, nutritional deficits, comorbidities, and pre-existing conditions may all contribute to HF vulnerability. Additionally, known stress-induced cytokine surges (e.g., IL-6, TNF-α) or microcirculatory impairment during acute COVID-19 could impact follicular health in a patient-specific manner.

**Genetic background**: It is plausible that certain genetic variants confer differential sensitivity of HFs to viral or stress-induced insults, though this remains to be elucidated.

Together, these factors might explain why, despite expression of entry proteins in the ORS and observed ex vivo permissiveness, TE remains a relatively rare complication. Our study contributes to identifying a potential molecular and cellular substrate for such cases, but larger clinical datasets and functional studies will be necessary to validate these findings in vivo and clarify their clinical relevance.

## Summary

Our study provides new insights into the pathophysiology of acute telogen effluvium (TE) in the context of COVID-19 and highlights a potential cellular mechanism linking SARS-CoV-2 exposure to hair follicle (HF) damage. We demonstrate that the main viral entry factors ACE2 and TMPRSS2 are co-expressed in the basal outer root sheath (ORS) cells of human HFs, and that viral nucleocapsid protein can be detected in the same compartment following ex vivo SARS-CoV-2 exposure. This spatial overlap suggests that SARS-CoV-2 proteins can reach and persist in epithelial stem or progenitor cells of the HF. Notably, the detection of apoptosis markers (CAS3 and TUNEL) in the same ORS cell population points to local stress responses that may contribute to hair follicle regression. Together, these findings support the hypothesis that SARS-CoV-2–induced follicular damage could initiate TE, even in the absence of confirmed productive viral replication. While our data suggest a potential interaction of viral proteins with epithelial stem or progenitor cells in the ORS, further analyses would be needed to determine whether this involves abortive or productive infection. While SARS-CoV-2–associated TE is typically self-limiting and reversible within three to six months, the condition can significantly affect quality of life and, in a minority of patients, may progress to chronic TE. The extent to which these symptoms are part of long COVID remains to be clarified. Our data contribute to a growing understanding of how viral infections may affect hair biology and underscore the need for further research into patient-specific vulnerability and therapeutic approaches.

## Materials and methods

### Post-mortem skin samples

Human skin samples were collected from one body donor donated to the Institute of Clinical Anatomy and Cell Analysis in Tübingen. The body donor gave its informed consent in accordance with the Declaration of Helsinki and approved by the Ethics Committee at the Medical Department of the University of Tübingen to use the cadaver for research purposes. Project number: 429/2022BO2, date of approval: 18.08.2022). The samples were taken within 8 h post-mortem.

### Isolation and culture of human plucked HFs

Human hair was plucked from two subjects (f: age 36, m: age 31). The quality of clearly visible white ORS from plucked HFs has been strictly controlled. The procedure of plucking human hair and further isolation of keratinocytes has been described already in detail^55^. In short: Hair was plucked under sterile conditions with a tweezer and immediately put into DMEM transportation medium with ambient temperature. After removing the hair shaft, the HF is put into a small flask with MEF-conditioned media until first keratinocytes appear. For further cultivation of primary keratinocytes EpiLife medium (Thermo Fisher) is used.

### Histological staining

10 μm longitudinal cryosections of plucked HFs were stained with haematoxylin and eosin (HE). First, samples were treated with filtered haematoxylin for 4 min, and eosin for 2 min with a washing step using running tap water. After performing an alcohol gradient (70%, 95%, 100%, 95%, 70%) with 30 s each, samples were placed into xylol for additional 30 s. The slides were mounted with DePEx mounting medium (VWR) and stored at room temperature.

### Immunofluorescence (IF) staining of cryosections and adherent cells

*IF of cryosections*: Skin-embedded HFs, plucked HFs and human kidney samples were fixed with 4% PFA + 10% sucrose for 15 min at room temperature followed by two washing steps with DPBS^−/−^ for each 5 min. Samples were mounted with Tissue-Tek (Sacura OCT), stored at -80 °C and later cut to 10 μm slices. For permeabilization, slices undergo an ethanol gradient each 30 Sect. (70%, 95%, 100%, 95% and 70%) and were blocked with skimmed milk blocking solution (10% NDS, 5% BSA, 0,1% Triton X, 4% skim milk) for 1 h at room temperature. Primary antibodies were diluted in blocking buffer and incubated overnight at 4 °C in a humidified chamber. Primary antibodies are listed in Supplemental Table T1. After 3 washing steps, secondary antibodies (Dαrb488, Dαrb546, Invitrogen, 1:1000) were diluted together with DAPI 1:1000 in DPBS^−/−^ and incubated for another 1 h at room temperature under exclusion of light. Sections were embedded with Mowiol mounting medium (Carl Roth).

**IF of adherent cells**: For adherent cells, the same protocol was used as for cryosections, with the exception that primary antibodies were incubated for 2 h at room temperature, followed by 3 washing steps with DPBS^−/−^ and further incubation with the secondary antibodies.

### Viral infection of human plucked HF

Two plucked hair follicles (HFs) were placed in each well of a 12-well culture plate and covered with a 13 mm coverslip to prevent floating. The wells were pre-coated for one hour with Matrigel: EpiLife (1:10) at 37 °C. Ex vivo infection experiments were performed in the BSL3 laboratory of the Virology Institute, UKT, Tübingen. HHFs were exposed to a clinical isolate of SARS-CoV-2 B.1 (D614G)^73^. No viral particles were added to the negative control (MOCK). 48 and 96 h post-infection (hpi) HFs were fixed for 30 min with 4% PFA and washed with PBS. Samples were further used for IF and TUNEL analysis. The protocol for immunofluorescence of cryosections was subsequently followed.

### TUNEL assay

To detect apoptosis in infected, plucked HFs, the Click-iT Plus TUNEL Assay protocol (Invitrogen, #C10619) was used. The blocking was performed using the same skimmed milk blocking solution as for the IF of cryosections. No Proteinase K was added. For the positive control, the slides were incubated with 1 unit of DNase I for 30 min.

### Microscopy

Bright field images were obtained using a PrimoVert light microscope (Zeiss). All immunofluorescence images were obtained using the Axio Imager M2 microscope with apotome (Zeiss) or Axio Scan. Z1 (Zeiss) and were analysed with the AxioVision software ZEN blue (Zeiss).

### RNA preparation and mRNA expression analysis

**RNA Preparation of human tissue**: HF (*n* = 3), keratinocyte (*n* = 3), as well as human kidney (*n* = 1), small intestine (*n* = 1), and testis (*n* = 1) samples were directly lysed in RLT buffer containing ß-mercaptoethanol (Carl Roth). RNA purification was performed using the RNeasy Micro Kit (all Qiagen) according to the manufacturer’s protocol.

### mRNA expression analysis of plucked HF

cDNA synthesis and preamplification was performed in one experimental setup, using 20 ng RNA together with SuperScript One-Step RT-PCR system with Platinum *Taq* DNA Polymerase and TE buffer (both Invitrogen) for 18 cycles in a StepOne Plus system (Thermo Fisher). Gene expression analysis was performed in a GE 96.96 Dynamic Array system (Fluidigm) using TaqMan Assays (Thermo Fisher), TaqMan Universal PCR Master Mix, no AmpErase UNG (Applied Biosystems) together with GE Sample Loading reagent (Fluidigm). Probes used for mRNA detection as well as RNA samples from human heart, liver, lung, and prostate were listed in Supplemental Table T2. Two technical measurements per RNA sample were analysed. Relative mRNA expression was calculated as a ratio of target gene concentration to the concentration of the housekeeping gene *GAPDH*. For all mRNA analysis, except the sex difference in the plucked HF (Supplemental Figure S1, K), the data of the male donor were used.

### Statistics of mRNA expression analysis

Quantitative PCR data were analysed using the 2⁻ΔCt method with *GAPDH* as endogenous reference gene. For each gene, mean 2⁻ΔCt values ± SD were calculated from *n* = 3 biological replicates (each measured in technical duplicates). Reference organ samples (testis, small intestine, kidney, heart, liver, lung, prostate) were analysed as single biological samples (*n* = 1) and are presented descriptively without statistical testing.

## Supplementary Information

Below is the link to the electronic supplementary material.

Supplementary Material 1

## Data Availability

The datasets generated and/or analysed during the current study are available from the corresponding author on reasonable request. Data are de-identified.
