# Supplementary material for: Ex vivo detection of SARS CoV 2 spike protein in human hair follicles and potential link to telogen effluvium
Source: Sci Rep. 2025 Nov 26;15:42019. doi: 10.1038/s41598-025-28909-3 (PMC12658035; doi:10.1038/s41598-025-28909-3)
Supplement: Supplementary file 1 — Supplementary Material 1 [file 41598_2025_28909_MOESM1_ESM.docx]

**SUPPLEMENTAL DATA**

1. *ACE2 and TMPRSS2 positive glandular cells*

Immunofluorescence staining of **ACE2** (**Supplemental Figure S1 A**) and **TMPRSS2** (**Supplemental Figure S1 B**) in longitudinal skin sections with embedded HF. Both proteins exhibit specific staining in **two distinct glandular types** in the skin. The magnified image sections clearly show that **ACE2** (**Supplemental Figure S1 A’**) and **TMPRSS2** (**Supplemental Figure S1 B’**) are positively expressed in **holocrine sebaceous glands**, which are located in close proximity to the hair shaft. Additionally, a strong expression is detected in the **coiled, merocrine sweat glands**, which are situated between the hair follicles in the dermis. Both proteins are produced in these glands (**Supplemental Figure S1 A’’, B’’)**.

1. *Expression of ORS marker KRT14*

To verify the different layers of the HF, staining with KRT14 was performed in addition to staining with ORS-specific marker KRT15 and ORS and companion layer-specific marker KRT6/75. KRT14 is a keratin that also occurs in the basal and suprabasal layers of ORS. Co-staining with ACE2 and TMPRRS2 in the cross section of skin-embedded HF confirmed that both ACE2 and TMPRRS2 are expressed in the ORS. In addition, a clear signal of TMPRS2 is found in the Henle’s layer of the IRS (**Supplemental Figure S1 C, D**).

1. *TMPRRS2 staining in longitudinal sections of skin-embedded human HFs*

Single TMRPSS2 protein expression can be detected in the ORS and in the Henle’s layer of the IRS. The signal in the ORS can best be seen in the middle area of the HF with a clearly visible root sheath (marked with orange arrows **Supplemental Figure S1 E**). In the transition zone between the papillary region and the ORS, where no ORS is developed and the surrounding sheath of the papilla is migrating into the IRS, TMPRSS2 can be found in the Henle’s layer of the IRS (marked with white arrows **Supplemental Figure S1 E**).

1. *Detection of endothelium in longitudinal sections of skin-embedded HFs*

In the HE staining of longitudinally sectioned HF (**Supplemental Figure S1 F**), capillaries filled with erythrocytes can be identified in the connective tissue layer, the dermal sheath, directly outward adjacent to the basal layer of the ORS (marked with black arrows in the enlarged **Supplemental Figure S1 F’**). In this histological staining, capillaries (marked with an orange arrow in the enlarged **Supplemental Figure S1 F’**) can also be found in the underlying dermis. Immunofluorescence staining with the lectin *Ulex Europaeus Agglutinin* (UEA1) (**Supplemental Figure S1 G**), an endothelial marker, also reveals smaller vessels in close proximity to the ORS of HFs (**Supplemental Figure S1 G’, G’’**).

1. *ACE2 and TMPRSS2 antibody verification in human kidney*

For verification of the primary and secondary antibodies used in this study, positive and negative controls have been performed. Human kidney was used as positive tissue for ACE2 and TMPRSS2 primary antibody and showed specific staining patterns (**Supplemental Figure S1 H, I**). To prove that fluorescence-labelled secondary antibodies do not have any unspecific binding patterns in the analysed tissue, negative controls with only the secondary antibody were performed (**Supplemental** **Figure S1 J**). No noticeable background was found in the analysed specimen.

1. *Sex difference in mRNA expression in human plucked HFs*

mRNA expression profile of *ACE2*, *TMPRSS2*, and three keratins (*KRT5, KRT14, KRT18*) in plucked human HFs from 2 donors with different sexes (**Supplemental Figure S1 K**). A difference could only be detected in *KRT5* mRNA expression, which is significantly higher expressed in female HFs (n=3, TTEST p<0,05).

1. *TUNEL expression in SARS-CoV-2-WT infected HFs*

In SARS-CoV-2 infected HFs, ACE2 and TMPRSS2, are both expressed in the basal, outermost layer of the HFs. The highest expression of ACE2 was detected in the ORS (**Supplemental Figure S2** **A**), while TMPRSS2 was weakly expressed in the ORS and showed its strongest expression in the Henle’s layer of the IRS (**Supplemental Figure S2** **B**). Single TUNEL staining for the detection of apoptosis in SARS-CoV-2-WT and Mock-infected HFs (**Supplemental Figure S2 C, D**). Main TUNEL expression can be detected in the outermost cell layer of the HF only in virus-infected HF. For the positive control of the TUNEL assay, the sections were first treated with DNase I for 30 minutes to induce double-strand breaks, followed by counterstaining of the nuclei with DAPI. TUNEL-positive cells can be detected in all layers of the HF (**Supplemental Figure 2 E, E'**).


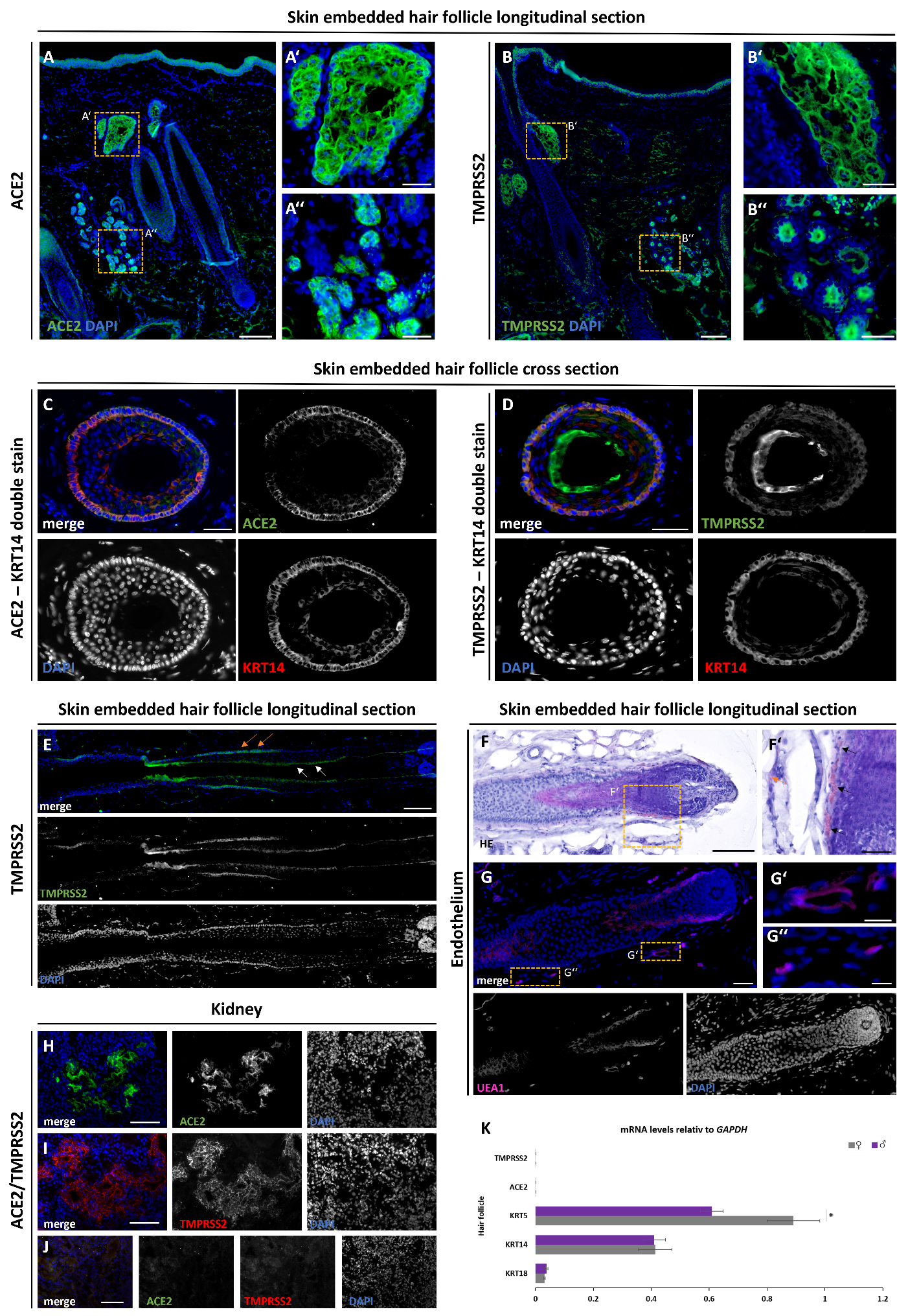


**Supplemental Figure S1: Staining of skin-embedded hair follicles (HFs), endothelium, kidney and sex difference in mRNA expression profile.** Immunofluorescence staining of ACE2 and TMPRSS2 in longitudinal skin sections (A, B) reveals their expression in two distinct gland types. Both proteins are present in holocrine sebaceous glands near the hair shaft (A’, B’) and in coiled merocrine sweat glands within the dermis (A’’, B’’). Co-staining of ACE2 (C, green) and TMPRSS2 (D, green) with outer root sheath (ORS) specific marker KRT14 (C, D, red) in cross sections of skin-embedded HFs. Single TMPRSS2 expression (E, green) in longitudinal section of a skin embedded HF. Orange arrows mark the region with well-developed ORS, white arrows mark the Henle’s layer in the transition zone between papilla and ORS. Histological HE staining (F) shows capillary in the dermal sheath right next to the ORS (marked in the highlighted magnification with black arrows, F’) and capillary in the dermis (marked in the highlighted magnification with orange arrow, F’). Immunofluorescence staining with the lectin UEA1 (G, magenta) also highlights smaller vessels in close proximity to the ORS of the HF (G’, G’’). Human kidney samples were used as positive control for the immunoreactivity of ACE2 (H, green) and TMPRSS2 (I, red) antibodies. Secondary antibody control for ACE2 (J, green) and TMPRSS2 (J, red) in human kidney samples. Cells were counterstained with nuclear marker DAPI (blue). Scale bar: 100 µm (C, D), 50 µm (A, B, D’, E, F, G, H), 20 µm (E’, E’’). Quantitative RNA expression profile of male (gray) and female (purple) plucked HF samples (K). In addition to *ACE2* and *TMPRSS2* probes, *KRT5*, *KRT14,* and *KRT18* have been measured. A significant difference in *KRT5* expression can be detected between both sexes. Number of single, independent experiments =3, TTEST p<0,05*.


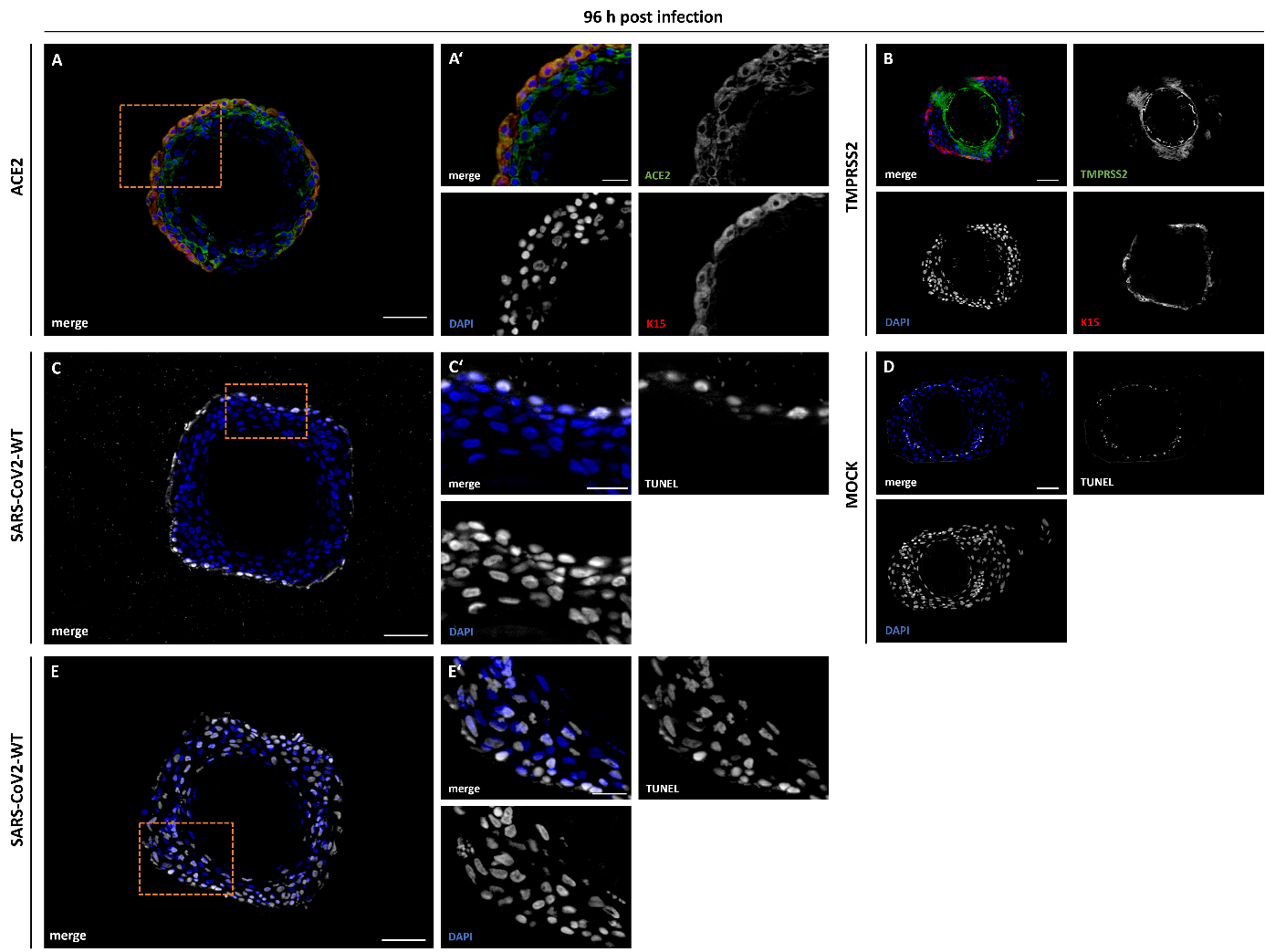


**Supplemental Figure S2: TUNEL expression in SARS-CoV-2-WT infected hair follicles (HFs).** Co-staining of K15 (red) and ACE2 (A, green), as well as TMPRSS2 (green, B) in SARS-CoV-2-WT infected HF. Main ACE2 expression can be detected in the ORS (A’), main TMPRSS2 expression can be detected in the ORS and the Henle’s layer of the inner root sheath (B). Single TUNEL staining (white) for the detection of apoptosis in SARS-CoV-2-WT (C) and Mock (D) infected HF. Main TUNEL expression can be detected in the outermost cell layer of the HF only in virus-infected HF, visible in the highlighted magnification (C’). For TUNEL positive control (E), slides were treated with DNAse I for 30 min. DNA strand breaks were detected by TUNEL staining, visible in the highlighted magnification (E’). Nuclear marker DAPI is shown in blue. Scale bar: 50 µm (A, B), 20 µm (A’, B’).

**Supplemental Table T1: Antibodies used in this study**

Table T1 summarizes the antibodies used for protein analysis

| **Name** | **Dilution** | **Supplier and ordering number** |
| --- | --- | --- |
| ACE2 | 1:100 | Abcam, #ab15348 |
| Cas 3 | 1:100 | Milipore, #AB3623 |
| Keratin 5 | 1:100 | Biolegend, #905501 |
| Keratin 6/75 | 1:50 | Santa Cruz, #Sc-166074 |
| Keratin 10 | 1:100 | Santa Cruz, #Sc-23877 |
| Keratin 14 | 1:100 | Thermo Fisher, #MA5-11599 |
| Keratin 15 | 1:50 | Santa Cruz, #Sc-47697 |
| Nucleocapsid | 1:1000 | Invitrogen, #MA1-7403 |
| TMPRSS2 | 1:150 | Abcam, #ab92323 |
| UEA1 | 1:400 | Vector, #DL-1068 |

**Supplemental Table T2: Probes used for RNA expression**

Table T2 summarizes the probes and RNA positive controls used for RNA expression analysis

| **Name** | **Number** | **Supplier** |
| --- | --- | --- |
| ACE2 | # Hs00222343_m1 | Thermo Fisher |
| Keratin 5 | # Hs00361185_m1 |  |
| Keratin 14 | # Hs00265033_m1 |  |
| Keratin 18 | # Hs02827483_g1 |  |
| GAPDH | # Hs99999905_m1 |  |
| TMPRSS2 | # H201122322_m1 |  |
|  | | |
| **Gene Expression Training Set**  Heart  Liver  Lung  Prostate | **100-8154**  #100-8159  #100-8160  #100-8161  #100-8162 | Fluidigm |
